# 244. Risk Factors Associated with Complications/Sequelae in Pediatric Patients with Osteomyelitis

**DOI:** 10.1093/ofid/ofab466.446

**Published:** 2021-12-04

**Authors:** Nancy Evelyn Aguilar Gómez, Aaron Espinosa Atri, Rafael Oscar Santamaría Vásquez, Alejandra Aquino Andrade, Isabel Medina Vera, Oscar Daniel Isunza Alonso, Leonor Patricia Saltigeral Simental

**Affiliations:** Instituto Nacional de Pediatría, Mexico, Distrito Federal, Mexico

## Abstract

**Background:**

Osteoarticular infections are serious invasive pathologies in the pediatric population. They have high morbidity, especially if antimicrobial treatment is inadequate and late. Based on pediatric series patients with osteomyelitis require prolonged antibiotic schemes, long stay and high hospital costs, multiple surgical procedures and develop short and long-term sequelae.

**Methods:**

A retrospective, observational, longitudinal and analytical study was conducted in patients under 17 years of age diagnosed with osteomyelitis at the National Institute of Pediatrics from January 2009 to January 2019. Demographic information, clinical presentation, microbiological, treatment and six-month follow-up were recorded.

**Results:**

A total of 109 patients were included, 57 (52%) males with median age of 98 (1-205) months with predominance in previous healthy (66%). By temporality, the chronic form predominated in 72%. The history of trauma was identified in 26% and fracture 19%. The most affected bone was femur 26%. Blood culture was performed in 55%, secretion culture in 52.2% with isolation in 56%. Methicillin-susceptible *Staphylococcus aureus* (MSSA) was the main agent identified. Complications occurred in 37%, the most frequent was surgical wound infection in 13% followed by fracture 11%. Risk factors for complications were chronic osteomyelitis RR 5.7 (CI 1.8-17.9), Sepsis/Shock RR 3.8 (CI 1.08-13-8) and MSSA infections RR 2.7 (CI 1.01-7.5); Risk factors for surgical site infection included initial fracture RR 3.5 (CI 1-11), local ulcer RR 4.2 (CI 1.3-13.06) and MSSA infection RR 5.9 (CI 1.8-19.4). Risk factors for limitation to movement included chronic osteomyelitis RR 4.87 (CI 1.6-14), fever RR 2.5 (CI 1.15-5.5), Sepsis/shock RR 5.3 (CI 1.3-20) (p 0.013) and MSSA infection RR 4.1 (CI 1.4-11.9).

**Conclusion:**

Osteomyelitis is still a health problem in our country. The diagnosis of osteomyelitis may be challenging as lack of suspicion often leads to delayed diagnosis. Knowledge of the risk factors for complications in pediatric patients could be useful to give early and proper antibiotic and surgical treatment. It is a priority to have a multidisciplinary team for the diagnosis and treatment of osteoarticular infections.

**Disclosures:**

**All Authors**: No reported disclosures

